# Hermite–Hadamard type inequalities for *n*-times differentiable and geometrically quasi-convex functions

**DOI:** 10.1186/s40064-016-2083-y

**Published:** 2016-04-26

**Authors:** Jun Zhang, Feng Qi, Gao-Chao Xu, Zhi-Li Pei

**Affiliations:** College of Computer Science and Technology, Jilin University, 130012 Changchun City, Jilin Province China; College of Computer Science and Technology, Inner Mongolia University for Nationalities, 028043 Tongliao City, Inner Mongolia Autonomous Region China; Department of Mathematics, School of Science, Tianjin Polytechnic University, 300387 Tianjin City, China; Institute of Mathematics, Henan Polytechnic University, 454010 Jiaozuo City, Henan Province China

**Keywords:** Hermite–Hadamard type inequality, geometrically quasi-convex function, Hölder integral inequality, Primary 26D15; Secondary 26A51, 26B12, 41A55, 49J52

## Abstract

By Hölder’s integral inequality, the authors establish some Hermite–Hadamard type integral inequalities for *n*-times differentiable and geometrically quasi-convex functions.

## Background

Let *I* be an interval on ${\mathbb {R}}=(-\infty ,\infty )$. A function $f:I\rightarrow {\mathbb {R}}$ is said to be convex if

1 $$f(\lambda x+(1-\lambda )y)\le \lambda f(x)+ (1-\lambda )f(y)$$

for $x,y\in I$ and $\lambda \in [0,1]$. If the inequality (1) reverses, then *f* is said to be concave on *I*.

A function $f:I\subseteq {\mathbb {R}}_+=(0,\infty )\rightarrow {\mathbb {R}}_+$ is said to be geometrically convex on *I* if

$$\begin{aligned} f\left(x^\lambda y^{1-\lambda }\right )\le \left[f(x)\right ]^\lambda \left[f(y)\right]^{1-\lambda } \end{aligned}$$

for $$x,y\in I$$ and $$\lambda \in [0,1]$$.

One of the most famous inequalities for convex functions is Hermite–Hadamard’s inequality: if $f:I\subseteq {\mathbb {R}}\rightarrow {\mathbb {R}}$ is convex on an interval *I* of real numbers and $a,b\in I$ with $a<b$, then

2 $$\begin{aligned} f\biggl (\frac{a+b}{2}\biggl )\le \frac{1}{b-a}\int _a^b f(x){{\mathrm{d}}}x \le \frac{f(a)+f(b)}{2}; \end{aligned}$$

if *f* is concave on *I*, then the inequality (2) is reversed.

We now collect several Hermite–Hadamard type integral inequalities as follows.

### **Theorem 1**

(Dragomir and Agarwal 1998) *Let*$f:I^\circ \subseteq {\mathbb {R}}\rightarrow {\mathbb {R}}$*be a differentiable mapping on*$I^\circ$*and*$a,b\in I^\circ$*with*$a<b$. *If*$|f'|$*is convex on* [*a*, *b*], *then*

$$\begin{aligned} \biggl |\frac{f(a)+f(b)}{2}-\frac{1}{b-a}\int _a^bf(x){{\mathrm{d}}}x\biggl |\le \frac{(b-a)\bigr [|f'(a)|+|f'(b)|\bigr ]}{8}. \end{aligned}$$

### **Theorem 2**

(Xi and Qi 2013) *Let*$f:I \subseteq {\mathbb {R}}_+\rightarrow {\mathbb {R}}$*be a differentiable function on*$I^\circ$*and*$a,b\in I^\circ$*with*$a<b$. *If*$|f'|$*is geometrically convex on* [*a*, *b*], *then*

$$\begin{aligned} \biggl |\frac{1}{\ln b-\ln a}\int _a^b\frac{f(x)}{x}{{\mathrm{d}}}x-f\bigl (\sqrt{a b}\,\bigr )\biggl |\le \frac{\ln b-\ln a}{4}\Bigl \{L\Bigl (\bigl [a|f'(a)|\bigr ]^{1/2},\bigl [b|f'(b)|\bigr ]^{1/2}\Bigr )\Bigl \}^2, \end{aligned}$$

*where*

$$\begin{aligned} L(u, v)=\frac{u-v}{\ln u-\ln v} \end{aligned}$$

*for*$u,v>0$ and $u\ne v$*is called the logarithmic mean.*

### **Theorem 3**

(Dragomir and Agarwal 1998) *Let*$f:I^\circ \subseteq {\mathbb {R}}\rightarrow {\mathbb {R}}$*be a differentiable mapping on*$I^\circ$*and*$a,b\in I^\circ$*with*$a<b$. *If*$|f'|^q$*for*$q\ge 1$*is convex on* [*a*, *b*], *then*

$$\begin{aligned} \biggl |\frac{f(a)+f(b)}{2}-\frac{1}{b-a}\int _a^bf(x){{\mathrm{d}}}x\biggl |\le \frac{b-a}{4}\biggl (\frac{|f'(a)|^q +|f'(b)|^q}{2}\biggr )^{1/q} \end{aligned}$$

*and*

$$\begin{aligned} \biggl |f\biggl (\frac{a+b}{2}\biggr )-\frac{1}{b-a}\int _a^bf(x){{\mathrm{d}}}x\biggl |\le \frac{b-a}{4}\biggl (\frac{|f'(a)|^q +|f'(b)|^q}{2}\biggr )^{1/q}. \end{aligned}$$

### **Theorem 4**

(Kirmaci 2004) *Let*$f:I\subseteq {\mathbb {R}}\rightarrow {\mathbb {R}}$*be differentiable on*$I^\circ$*and*$a,b\in I$*with*$a<b$. *If*$|f'|^{p/(p-1)}$*for*$p>1$*is convex on* [*a*, *b*], *then*

$$\begin{aligned} \biggl |f\biggl (\frac{a+b}{2}\biggl )-\frac{1}{b-a}\int _a^bf(x){{\mathrm{d}}}x\biggl |\le \frac{b-a}{16}\biggl (\frac{4}{p+1}\biggl )^{1/p} \biggl \{\Bigr [|f'(a)|^{p/(p-1)}\\ +3|f'(b)|^{p/(p-1)}\Bigl ]^{1-1/p} +\Bigr [3|f'(a)|^{p/(p-1)}+|f'(b)|^{p/(p-1)}\Bigl ]^{1-1/p}\biggl \}. \end{aligned}$$

Corresponding to the concept of geometrically convex functions, the geometrically quasi-convex functions were introduced in Qi and Xi (2014) as follows.

### **Definition 1**

(*Definition 2.1 Qi and Xi *2014) A function $f:I\subseteq {\mathbb {R}}_+\rightarrow {\mathbb {R}}_0=[0,\infty )$ is said to be geometrically quasi-convex function on *I* if

$$\begin{aligned} f\bigl (x^\lambda y^{1-\lambda }\bigr )\le \sup \{f(x),f(y)\} \end{aligned}$$

for $$x,y\in I$$ and $$\lambda \in [0,1]$$.

In Qi and Xi (2014), some integral inequalities of Hermite–Hadamard type for geometrically quasi-convex functions were established.

In recent years, some other kinds of Hermite–Hadamard type inequalities were generated. For more systematic information, please refer to Bai et al. (2012), Pearce and Pečarić (2000), Pečarić and Tong (1991), Wang and Qi (2013), Wang et al. (2012), Xi et al. (2012) and related references therein.

The aim of this paper is to find more integral inequalities of Hermite–Hadamard type for *n*-times differentiable and geometrically quasi-convex functions.

## A Lemma

In order to obtain our main results, we need the following Lemma.

### **Lemma 1**

(Wang and Shi 2016) *For*$n\in {\mathbb {N}}$, *let*$f:I\subseteq {\mathbb {R}}_+\rightarrow {\mathbb {R}}$*be an*-*times differentiablefunction on*$I^\circ$*and*$a,b\in I$*with*$a< b$. *If*$f^{(n)}\in L_1([a,b])$, *then*

$$\begin{aligned}&\sum _{k=1}^{n}\frac{(-1)^{k-1}}{k!}\bigl [b^kf^{(k-1)}(b)-a^{k}f^{(k-1)}(a)\bigr ] -\int _a^{b}f(x){{\mathrm{d}}}x \\&\qquad = \frac{(-1)^{n-1}(\ln b-\ln a)}{n!}\int _0^1a^{(n+1)t}b^{(n+1)(1-t)}f^{(n)}\bigl (a^tb^{1-t}\bigr ) {{\mathrm{d}}}t. \end{aligned}$$

### *Remark 1*

Under the conditions of Lemma 1, taking $n=1$, we obtain

$$\begin{aligned} bf(b)-af(a)-\int _a^{b}f(x){{\mathrm{d}}}x = (\ln b-\ln a\bigr )\int _0^1a^{2t}b^{2(1-t)}f'\bigl (a^tb^{1-t}\bigr ) {{\mathrm{d}}}t, \end{aligned}$$

which can be found in Zhang et al. (2013).

## Inequalities for geometrically quasi-convex functions

Now we start out to establish some new Hermite–Hadamard type inequalities for *n*-times differentiable and geometrically quasi-convex functions.

### **Theorem 5**

*For *$n\in {\mathbb {N}}$*, suppose that*$f:I\subseteq {\mathbb {R}}_+\rightarrow {\mathbb {R}}$*is a n-times differentiable function on *$I^\circ$*, that*$f^{(n)}\in L_1([a,b])$*, and that *$a,b\in I$* with*$a<b$*. If*$\bigl |f^{(n)}\bigr |^q$*is geometrically quasi-convex on [a, b] for *$q\ge 1$*, then*

$$\begin{aligned}&\Biggl |\sum _{k=1}^{n}\frac{(-1)^{k-1}}{k!}\bigl [b^kf^{(k-1)}(b)-a^{k}f^{(k-1)}(a)\bigr ] -\int _{a}^{b}f(x){{\mathrm{d}}}x \Biggl |\\&\quad \le \frac{(\ln b-\ln a)}{n!}L\bigl (a^{n+1},b^{n+1}\bigr )\sup \bigl \{\bigr |f^{(n)}(a)\bigr |,\bigr |f^{(n)}(b)\bigr |\bigr \}. \end{aligned}$$

### *Proof*

By the geometric quasi-convexity of $\bigl |f^{(n)}\bigr |^q$, Lemma 1, and Hölder’s inequality, one has

$$\begin{aligned}&\Biggl |\sum _{k=1}^{n}\frac{(-1)^{k-1}}{k!}\bigl [b^kf^{(k-1)}(b)-a^{k}f^{(k-1)}(a)\bigr ] -\int _{a}^{b}f(x){{\mathrm{d}}}x \Biggl |\\&\quad\le \frac{\ln b-\ln a}{ n!}\int _0^1a^{(n+1)t}b^{(n+1)(1-t)}\Bigr |f^{(n)}\bigl (a^{t}b^{1-t}\bigr )\Bigr |{{\mathrm{d}}}t\\&\quad\le \frac{\ln b-\ln a}{ n!}\biggl [\int _0^1a^{(n+1)t}b^{(n+1)(1-t)}{{\mathrm{d}}}t\biggr ]^{1-1/q}\\&\quad \times \biggl \{\int _0^1a^{(n+1)t}b^{(n+1)(1-t)}\sup \bigl \{\bigr |f^{(n)}(a)\bigr |^q,\bigr |f^{(n)}(b)\bigr |^q\bigr \} {{\mathrm{d}}}t\biggr \}^{1/q}\\&\quad= \frac{(\ln b-\ln a)L\bigl (a^{n+1}, b^{n+1}\bigr )}{ n!}\sup \bigl \{\bigr |f^{(n)}(a)\bigr |,\bigr |f^{(n)}(b)\bigr |\bigr \}. \end{aligned}$$

Theorem 5 is thus proved.$\square$

### **Corollary 1**

*Under the assumptions of Theorem* 5, *if*$q=1$, *then*

$$\begin{aligned}&\Biggl |\sum _{k=1}^{n}\frac{(-1)^{k-1}}{k!}\bigl [b^kf^{(k-1)}(b)-a^{k}f^{(k-1)}(a)\bigr ] -\int _{a}^{b}f(x){{\mathrm{d}}}x \Biggl |\\&\quad \le \frac{\ln b-\ln a}{n!}L\bigl (a^{(n+1)},b^{(n+1)}\bigr )\sup \bigl \{\bigr |f^{(n)}(a)\bigr |,\bigr |f^{(n)}(b)\bigr |\bigr \}. \end{aligned}$$

### **Theorem 6**

*For*$n\in {\mathbb {N}}$, *suppose that*$f:I\subseteq {\mathbb {R}}_+\rightarrow {\mathbb {R}}$*is an*-*times differentiablefunction on*$I^\circ$, *that*$f^{(n)}\in L_1([a,b])$, *and that*$a,b\in I$*with*$a<b$. *If*$\bigl |f^{(n)}\bigr |^q$*is geometrically quasi-convex on* [*a*, *b*] *for*$q>1$, *then*

$$\begin{aligned}&\Biggl |\sum _{k=1}^{n}\frac{(-1)^{k-1}}{k!}\bigl [b^kf^{(k-1)}(b)-a^{k}f^{(k-1)}(a)\bigr ] -\int _{a}^{b}f(x){{\mathrm{d}}}x \Biggl |\le \frac{\ln b-\ln a}{n!}\\&\quad \times \Bigl [L\Bigl (a^\frac{q(n+1)-m}{q-1}, b^\frac{q(n+1)-r}{q-1}\Bigr )\Bigr ]^{1-1/q}\bigl [L\bigl (a^m, b^r\bigr )\bigr ]^{1/q} \sup \bigl \{\bigr |f^{(n)}(a)\bigr |,\bigr |f^{(n)}(b)\bigr |\bigr \} \end{aligned}$$

*for*$$0\le m, r\le (n+1)q$$.

### *Proof*

From the geometric quasi-convexity of $\bigl |f^{(n)}\bigr |^q$, Lemma 1, and Hölder’s inequality, we have

$$\begin{aligned}&\Biggl |\sum _{k=1}^{n}\frac{(-1)^{k-1}}{k!}\bigl [b^kf^{(k-1)}(b)-a^{k}f^{(k-1)}(a)\bigr ] -\int _{a}^{b}f(x){{\mathrm{d}}}x \Biggl |\\&\le \frac{\ln b-\ln a}{ n!}\int _0^1a^{(n+1)t}b^{(n+1)(1-t)}\bigl |f^{(n)}\bigl (a^{t}b^{1-t}\bigr )\bigr |{{\mathrm{d}}}t\\&\le \frac{\ln b-\ln a}{ n!}\biggl [\int _0^1a^{[q(n+1)-m]t/(q-1)}b^{[q(n+1)-r](1-t)/(q-1)}{{\mathrm{d}}}t\biggr ]^{1-1/q}\\&\quad \times \biggl \{\int _0^1a^{mt}b^{r(1-t)}\sup \bigl \{\bigr |f^{(n)}(a)\bigr |^q,\bigr |f^{(n)}(b)\bigr |^q\bigr \} {{\mathrm{d}}}t\biggr \}^{1/q}\\&= \frac{\ln b-\ln a}{n!}\Bigl [L\Bigr (a^\frac{q(n+1)-m}{q-1}, b^\frac{q(n+1)-r}{q-1}\Bigl )\Bigr ]^{1-1/q}\bigl [L\bigl (a^m, b^r\bigr )\bigr ]^{1/q}\\&\quad \times \sup \bigl \{\bigr |f^{(n)}(a)\bigr |,\bigr |f^{(n)}(b)\bigr |\bigr \}. \end{aligned}$$

The proof of Theorem 6 is complete. $\square$

### **Corollary 2**

*Under the conditions in Theorem* 6,

1. *if*$$m=r=0$$, *then*
  $$\begin{aligned}&\Biggl |\sum _{k=1}^{n}\frac{(-1)^{k-1}}{k!}\bigl [b^kf^{(k-1)}(b)-a^{k}f^{(k-1)}(a)\bigr ] -\int _{a}^{b}f(x){{\mathrm{d}}}x \Biggl |\\&\quad \le \frac{\ln b-\ln a}{n!}\Bigl [L\Bigl (a^\frac{q(n+1)}{q-1}, b^\frac{q(n+1)}{q-1}\Bigr )\Bigr ]^{1-1/q}\sup \bigl \{\bigr |f^{(n)}(a)\bigr |,\bigr |f^{(n)}(b)\bigr |\bigr \}; \end{aligned}$$
2. *if*$$m=r=q(n+1)$$, *then*
  $$\begin{aligned}&\Biggl |\sum _{k=1}^{n}\frac{(-1)^{k-1}}{k!}\bigl [b^kf^{(k-1)}(b)-a^{k}f^{(k-1)}(a)\bigr ] -\int _{a}^{b}f(x){{\mathrm{d}}}x \Biggl |\\&\quad \le \frac{\ln b-\ln a}{ n!}\bigl [L\bigl (a^{q(n+1)}, b^{q(n+1)}\bigr )\bigr ]^{1/q}\sup \bigl \{\bigr |f^{(n)}(a)\bigr |,\bigr |f^{(n)}(b)\bigr |\bigr \}; \end{aligned}$$
3. *if*$m=0$*and*$r=q(n+1)$, *then*
  $$\begin{aligned}&\Biggl |\sum _{k=1}^{n}\frac{(-1)^{k-1}}{k!}\bigl [b^kf^{(k-1)}(b)-a^{k}f^{(k-1)}(a)\bigr ] -\int _{a}^{b}f(x){{\mathrm{d}}}x \Biggl |\le \frac{\ln b-\ln a}{n!}\\&\quad \times \Bigl [L\Bigl (a^\frac{q(n+1)}{q-1}, 1\Bigr )\Bigr ]^{1-1/q}\bigl [L\bigl (1, b^{q(n+1)}\bigr )\bigr ]^{1/q}\sup \bigl \{\bigr |f^{(n)}(a)\bigr |,\bigr |f^{(n)}(b)\bigr |\bigr \}; \end{aligned}$$
4. *if*$m=n+1$*and*$r=q(n+1)$, *then*
  $$\begin{aligned}&\Biggl |\sum _{k=1}^{n}\frac{(-1)^{k-1}}{k!}\bigl [b^kf^{(k-1)}(b)-a^{k}f^{(k-1)}(a)\bigr ] -\int _{a}^{b}f(x){{\mathrm{d}}}x \Biggl |\le \frac{\ln b-\ln a}{n!}\\&\quad \times \bigl [L\bigl (a^{n+1}, 1\bigr )\bigr ]^{1-1/q}\bigl [L\bigl (a^{n+1}, b^{q(n+1}\bigr )\bigr ]^{1/q}\sup \bigl \{\bigr |f^{(n)}(a)\bigr |,\bigr |f^{(n)}(b)\bigr |\bigr \}; \end{aligned}$$
5. *if*$m=q(n+1)$*and*$r=0$, *then*
  $$\begin{aligned}&\Biggl |\sum _{k=1}^{n}\frac{(-1)^{k-1}}{k!}\bigl [b^kf^{(k-1)}(b)-a^{k}f^{(k-1)}(a)\bigr ] -\int _{a}^{b}f(x){{\mathrm{d}}}x \Biggl |\le \frac{\ln b-\ln a}{n!}\nonumber \\&\quad \times \Bigl [L\Bigl (1, b^\frac{q(n+1)}{q-1}\Bigr )\Bigr ]^{1-1/q}\bigl [L\bigl (a^{q(n+1}, 1\bigr )\bigr ]^{1/q}\sup \bigl \{\bigr |f^{(n)}(a)\bigr |,\bigr |f^{(n)}(b)\bigr |\bigr \}; \end{aligned}$$
6. *if*$m=q(n+1)$*and*$r=n+1$, *then*
  $$\begin{aligned}&\Biggl |\sum _{k=1}^{n}\frac{(-1)^{k-1}}{k!}\bigl [b^kf^{(k-1)}(b)-a^{k}f^{(k-1)}(a)\bigr ] -\int _{a}^{b}f(x){{\mathrm{d}}}x \Biggl |\le \frac{\ln b-\ln a}{n!}\\&\quad \times \bigl [L\bigl (1, b^{n+1}\bigr )\bigr ]^{1-1/q}\bigl [L\bigl (a^{q(n+1)}, b^{n+1}\bigr )\bigr ]^{1/q} \sup \bigl \{\bigr |f^{(n)}(a)\bigr |,\bigr |f^{(n)}(b)\bigr |\bigr \}. \end{aligned}$$

## Conclusion

Our main results in this paper are those integral inequalities of Hermite–Hadamard type in Theorems 5 and 6 and Corollaries 1 and 2.
